# Research on sleep disorders and related risk factors among healthcare workers from Fujian province supporting Hubei province during the COVID-19 pandemic

**DOI:** 10.3389/fpsyg.2024.1390410

**Published:** 2024-07-31

**Authors:** Wu Yongxi, Lin Zexin, Lin Yiqi, Lin Qianwen, Xie Xiaoliang, Wei Shichao

**Affiliations:** ^1^Fujian Provincial Hospital, Shengli Clinical Medical College of Fujian Medical University, Fuzhou, Fujian, China; ^2^Fuzhou University Affiliated Provincial Hospital, Fuzhou, Fujian, China; ^3^Nanjing Brain Hospital, Affiliated Hospital of Nanjing Medical University, Nanjing, Jiangsu, China

**Keywords:** COVID-19 pandemic, sleep disorders, healthcare, Fujian province, COVID-19

## Abstract

**Objective:**

To explore the impact of COVID-19 on the sleep of healthcare workers from Fujian Province supporting Hubei Province and its related risk factors.

**Methods:**

A cross-sectional, anonymous, self-reported online questionnaire survey was conducted among all participants. The questionnaire consisted of five parts: sociodemographic characteristics and COVID-19 epidemic-related factors, Pittsburgh Sleep Quality Index (PSQI), Epworth Sleepiness Scale (ESS), Morningness-Eveningness Questionnaire-5 (MEQ-5), and 12-item General Health Questionnaire (GHQ-12).

**Results:**

Among 552 participants, 203 (36.8%) had a PSQI score > 7, indicating the presence of sleep disorders. *Logistic* regression analysis revealed that sleep disorders were independently associated with a history of previously diagnosed sleep disorders (OR 6.074, 95% *CI* 2.626–14.049, *P* < 0.001), rotating night shifts > 3 times per week (OR 3.089, 95% *CI* 1.650–5.781, *P* < 0.001), using electronic devices before sleep >1 h (OR 1.685, 95% *CI* 1.131–2.511, *P* = 0.010), concern about contracting COVID-19 (OR 1.116, 95% *CI* 1.034–1.204, *P* = 0.005), perception of societal support for supporting healthcare workers in Hubei (OR 0.861,95% *CI* 0.744–0.998, *P* = 0.047) (OR 0.861, 95% *CI* 0.744–0.998, *P* = 0.047), non-medical staff (OR 0.257, 95% *CI* 0.067–0.987, *P* = 0.048), ESS score (OR 1.068, 95% *CI* 1.018–1.121, *P* = 0.007), and GHQ-12 score (OR 1.511, 95% *CI* 1.281–1.782, *P* < 0.001).

**Conclusion:**

Sleep disorders were highly prevalent among healthcare workers from Fujian Province supporting Hubei Province during the COVID-19 pandemic. Risk factors for sleep disorders included a history of previously diagnosed sleep disorders, rotating night shifts > 3 times per week, using electronic devices before sleep >1 h, excessive concern about contracting COVID-19, and poorer psychological health. Higher perceived societal support and understanding of support for healthcare workers supporting Hubei were associated with a reduced risk of sleep disorders, as was being non-medical staff. Providing more sleep hygiene education and psychological health services for frontline healthcare workers is necessary.

## 1 Introduction

Since the first case of novel coronavirus pneumonia (COVID-19) was reported in late December 2019, COVID-19 has continued to spread in most countries around the world. COVID-19 is characterized by high efficiency of transmission among individuals, high risk of infection among medical personnel, and a high rate of severe illness, which has had a significant impact on global healthcare. Following the outbreak, 42,000 healthcare workers responded to the national call to support Hubei, among whom 1,393 were from Fujian Province.

Previous studies have shown that patients who survive acute infectious diseases (such as SARS) often experience mental health problems such as anxiety, depression, and post-traumatic stress disorder (Wu et al., 2005). This virus outbreak has spread rapidly, with a high risk of infection, and medical personnel often need to work in enclosed environments wearing masks and isolation gowns. While this reduces the risk of virus infection, it increases their anxiety and fear (Wang C. et al., 2020). Additionally, during the Chinese New Year holiday, they were unable to reunite with their families, which posed a great threat to the physical and mental health of healthcare workers. Adverse emotions can affect sleep, and good sleep can help maintain optimal immune function (Lange et al., 2010).

Sleep disorders are particularly critical for healthcare workers, as they are associated with negative outcomes such as impaired cognitive function, increased risk of errors, and overall poor health. Studies have indicated that the mental health of healthcare workers during the COVID-19 pandemic is significantly impacted, leading to increased sleep disturbances. For example, Esmaeilnia discuss the psychomotor and physiological impacts on individuals post-COVID-19 recovery, highlighting the importance of addressing these issues for healthcare workers (Esmaeilnia et al., 2023). Furthermore, Keyvanfar demonstrate a strong association between sleep quality and mental health among medical students, which can be extrapolated to healthcare workers under pandemic conditions (Keyvanfar et al., 2022). Rassolnia and Nobari explore the impact of socio-economic status and physical activity on psychological well-being and sleep quality, emphasizing the broader factors influencing health during the pandemic (Rassolnia and Nobari, 2024).

Therefore, conducting various forms of sleep and psychological surveys is beneficial for enhancing people’s psychological and neural immunity to COVID-19 and safeguarding their physical and mental health. This study investigated the sleep and psychological impact of the COVID-19 epidemic on healthcare workers from Fujian Province who supported Hubei. At the same time, their lifestyle and sleep habits were investigated to understand the risk factors for sleep disorders. Our research aims to fill existing gaps by providing a detailed analysis of sleep disorders among healthcare workers and suggesting specific interventions to mitigate these risks.

## 2 Materials and methods

### 2.1 Study population

**FIGURE 1:**
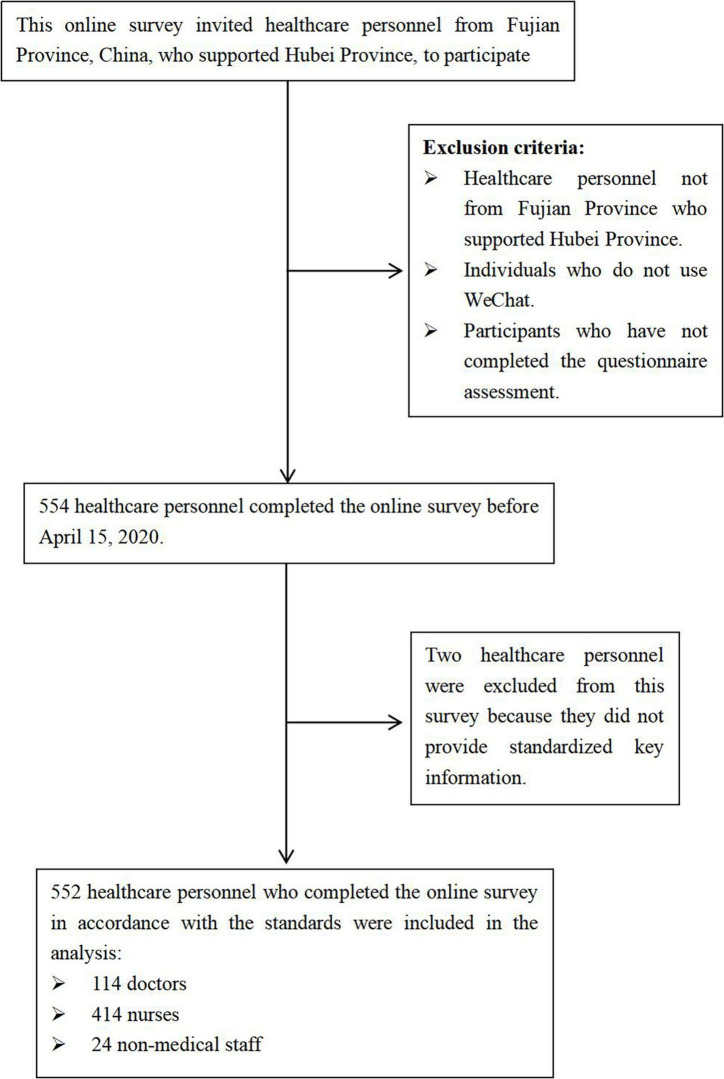
Flowchart of participant recruitment.

A cross-sectional study was conducted anonymously from March 19, 2020, to April 15, 2020, in Hubei Province, China, using an online questionnaire survey via the social media platform. We Chat and the Questionnaire Star software. The participants were healthcare workers from Fujian Province who supported Hubei, including doctors, nurses, and other non-medical staff (such as healthcare system administrators, drivers of national emergency rescue teams, etc.). The questionnaire was completed by participants using their own smartphones. The entire questionnaire took approximately 15 to 30 min to complete. Prior to the survey, the purpose of the study was explained to each participant by the researchers. We provided clear instructions and definitions for key terms within the questionnaire to minimize misunderstandings and ensure that participants interpreted the questions consistently. We ensured that responses were anonymous and confidential and all participants were informed and consented to participate in the survey. The detailed process of this study is shown in Figure 1, and the study was approved by the Ethics Committee of Fujian Provincial Hospital.

### 2.2 Research methods

#### 2.2.1 Survey methods

Social demographic data, including gender, age, marital status, etc., were collected through an online questionnaire survey. Additionally, factors related to the COVID-19 pandemic were collected, such as participants’ job roles, locations of support work, and the frequency of night shifts per week.

#### 2.2.2 Survey instruments

In our study, the Pittsburgh Sleep Quality Index (PSQI) was used to assess sleep quality, primarily evaluating the quality of sleep over the past month (Buysse et al., 1989). Studies from China have confirmed that when the PSQI score is > 7 points, with a cutoff value of 98% sensitivity and 90% specificity (kappa = 0.89, *P* < 0.01), it is suitable for the Chinese population (Liu and Tang, 1996). In our study, a PSQI score > 7 points was also used to determine sleep disorders.

Daytime sleepiness was assessed using the Epworth Sleepiness Scale (ESS), developed by Johns et al., to semi-objectively assess participants’ recent daytime sleepiness over the past few months. The scale consists of 8 items, each scored from 0 to 3, with a total score ranging from 0 to 24. A cutoff score of 10 was used in our study to indicate daytime sleepiness (Johns, 1991).

Sleep rhythm assessment was conducted using the Morningness-Eveningness Questionnaire-5 (MEQ-5), which comprises 5 items from the MEQ-19 (items 1, 7, 10, 18, 19), with a total score ranging from 4 to 25. The cutoff points recommended by Adan and Almirall (1991) were used: 4–7 points for Definitely Evening (DE), 8–11 points for Moderately Evening (ME), 12–17 points for Neutral (N), 18–21 points for Moderately Morning (MM), and 22–25 points for Definitely Morning (DM).

The assessment of mental health status was measured using the 12-item General Health Questionnaire (GHQ-12) (Goldberg et al., 1997). The questionnaire consists of 12 items, each evaluated by 4 indicators, with a total score ranging from 0 to 12 using the bimodal scoring method (0-0-1-1), which is one of the widely recognized methods for better reflecting results (Montazeri et al., 2003). Higher scores indicate a higher degree of mental health disorder, with a cutoff value of 3/4 points being optimal (Goldberg et al., 1997). In our study, a score of ≥ 4 points was used to indicate the presence of mental health problems.

#### 2.3 Statistical analysis

Statistical analysis was performed using SPSS 22.0 software. Count data were presented as percentages (%), and intergroup comparisons were conducted using the chi-square test. Normally distributed metric data were expressed as mean ± standard deviation. For metric data not conforming to a normal distribution, the median (M) and quartiles were used, and intergroup comparisons were made using the Mann-Whitney U test. Multifactorial logistic regression analysis was employed to screen for risk factors of sleep disorders. Differences were considered statistically significant at a threshold of *P* < 0.05.

## 3. Results

### 3.1 Comparison of sociodemographic data and clinical data related to COVID-19

A total of 552 healthcare workers were included in the study, comprising 149 males (27.0%) and 403 females (73.0%). There were statistically significant differences between the two groups in terms of job status, frequency of night shifts per week, history of previously diagnosed sleep disorders, and family history of sleep disorders (*P* < 0.05). However, there were no statistically significant differences between the two groups in terms of gender, age, marital status, location of support work, total daily working hours, number of working days per week, and duration of support work (*P* > 0.05). See Table 1 for details.

**TABLE 1:** Comparison of sociodemographic data and clinical data related to COVID-19.

Item	PSQI ≤ 7 (*n* = 349)	PSQI>7 (*n* = 203)	χ^2^/Z	*P-*value
Gender [Male, *n* (%)]	100 (28.7)	49 (24.1)	1.328	0.249
Age [years, (P25, P75)]	33.00 (30.00, 38.00)	33.00 (29.00, 38.00)	−0.017^△^	0.987
**Marital Status [*n* (%)]**			1.093	0.779
Unmarried	106 (30.4)	65(32.0)		
Married	226 (64.7)	131 (64.5)		
Divorced	16 (4.6)	7 (3.4)		
Other	1 (0.3)	0 (0.0)		
Job Status [n (%)]			6.076	0.048
Doctor	77 (22.1)	37 (18.2)		
Nurse	252 (72.2)	162 (79.8)		
Non-medical staff	20 (5.7)	4 (2.0)		
Location of Support Work [n (%)]			3.651	0.161
Cabin hospital ward	68 (19.5)	31 (15.3)		
Designated hospital ward	270 (77.3)	160 (78.8)		
Other	11 (3.2)	12 (5.9)		
Frequency of Night Shifts per Week [n (%)]			24.808	<0.001
1 time	74 (21.2)	38 (18.7)		
2 times	135 (38.7)	54 (26.6)		
3 times	95 (27.2)	51 (25.1)		
> 3 times	45 (12.9)	60 (29.6)		
Total Daily Working Hours [hours, (P25, P75)]	8.00 (7.00,9.50)	8.00 (7.50,10.00)	−1.441^△^	0.150
Number of Working Days per Week [days, (P25, P75)]	5.00 (4.00,6.00)	5.00 (4.00,6.00)	−0.703^△^	0.482
**Duration of Support Work [*n* (%)]**			1.439	0.487
<2 weeks	2 (0.6)	0 (0.0)		
2–4 weeks	17 (4.9)	8 (3.9)		
>4 weeks	330 (94.5)	195 (96.1)		
History of Previously Diagnosed Sleep Disorders [*n* (%)]	12 (3.4)	22 (10.8)	12.156	<0.001
Family History of Sleep Disorders [*n* (%)]	11 (3.2)	17 (8.4)	7.270	0.007

△ represents the Z value for the Mann-Whitney U test, all others represent χ^2^ values. PSQI, Pittsburgh Sleep Quality Index.

### 3.2 Comparison of daily habits and sociopsychological factors related to COVID-19

Comparison of daily habits and sociopsychological factors related to COVID-19 between the two groups showed significant differences (*P* < 0.05). These differences were observed in the duration of using electronic devices for over 1 h before bedtime, concerns about contracting COVID-19, confidence in recovery if infected, perception of societal support for healthcare workers supporting Hubei, and family support for epidemic prevention work. However, there were no statistically significant differences (*P* > 0.05) in the types and purposes of using electronic devices before bedtime, daytime napping duration, and the level of concern about family members contracting COVID-19. See Table 2 for details.

**TABLE 2:** Comparison of participants’ daily habits and sociopsychological factors related to COVID-19.

Item	PSQI ≤ 7 (*n* = 349)	PSQI>7 (*n* = 203)	χ^2^/Z	*P*-value
Using electronic devices > 1 h before sleep [*n* (%)]	124 (35.5)	111 (54.7)	19.251	<0.001
**Types of electronic devices used before sleep**				
– Computer [*n* (%)]	22 (6.3)	13 (6.4)	0.002	0.963
– Smartphone [*n* (%)]	331 (94.8)	197 (97.0)	1.496	0.221
– Tablet [*n* (%)]	21 (6.0)	11 (5.4)	0.084	0.772
– No electronic device [*n* (%)]	16 (4.6)	3 (1.5)	3.727	0.054
**Purposes of using electronic devices before sleep**				
– Playing games [*n* (%)]	40 (11.5)	27 (13.3)	0.407	0.523
– Reading novels/news [*n* (%)]	211 (60.5)	133 (65.5)	1.399	0.237
– Watching videos/movies [*n* (%)]	209 (59.9)	132 (65.0)	1.436	0.231
– Social chatting [*n* (%)]	213 (61.0)	128 (63.1)	0.222	0.637
– Other purposes [*n* (%)]	35 (10.0)	26 (12.8)	1.009	0.315
Daytime napping duration			4.775	0.311
– 0 min [*n* (%)]	67 (19.2)	53 (26.1)		
- < 30 min [*n* (%)]	23 (6.6)	15 (7.4)		
– 30∼60 min [*n* (%)]	187 (53.5)	93 (45.8)		
– 60∼120 min [*n* (%)]	70 (20.1)	40 (19.7)		
- > 120 min [*n* (%)]	2 (0.6)	2 (1.0)		
Feeling competent in current work tasks [points, (P25, P75)]	8.00 (8.00,10.00)	8.00 (7.00,10.00)	−1.679^△^	0.093
Concern about contracting COVID-19 [points, (P25, P75)]	5.00 (2.00,7.00)	6.00 (5.00,8.00)	−4.688^△^	<0.001
Confidence in recovery if infected [points, (P25, P75)]	9.00 (8.00,10.00)	8.00 (7.00,10.00)	−2.597^△^	0.009
Concern about family contracting COVID-19 [points, (P25, P75)]	5.00 (2.00,8.00)	6.00 (3.00,8.00)	−1.847^△^	0.065
Perception of societal support for healthcare workers supporting Hubei [points, (P25, P75)]	10.00 (9.00,10.00)	10.00 (8.00,10.00)	−2.143^△^	0.032
Family support for epidemic prevention work			6.066	0.048
– Support [*n* (%)]	336 (96.3)	186 (91.6)		
– Not support [*n* (%)]	1 (0.3)	3 (1.5)		
– Inconsistent opinions [*n* (%)]	12 (3.4)	14 (6.9)		

△ represents the Z value for the Mann-Whitney U test, all others represent χ^2^ values. PSQI, Pittsburgh Sleep Quality Index.

### 3.3 Comparison of participants’ sleep quality and sleep-related psychological scale data

A total of 203 participants (36.8%) were found to have a PSQI score > 7, indicating the presence of sleep disorders. The PSQI total score for the sleep disorder group was 10.00 (9.00, 12.00) points, while it was 4.00 (3.00, 6.00) points for the non-sleep disorder group. The sleep disorder group exhibited significantly higher PSQI total scores, as well as scores for sleep onset, sleep duration, sleep efficiency, sleep disturbances, sleep medication usage, daytime dysfunction, and overall sleep quality compared to the non-sleep disorder group, with statistical significance (*P* < 0.001). Significant differences were observed between the two groups in ESS scores, GHQ-12 scores, and MEQ-5 classification (*P* < 0.001). The sleep disorder group had higher ESS and GHQ-12 scores, and a greater proportion of individuals with eveningness chronotype (definite evening and moderate evening) compared to the non-sleep disorder group. Regarding daytime sleepiness, 45 individuals (22.2%) in the sleep disorder group experienced daytime sleepiness, which was higher than the 38 individuals (10.9%) in the non-sleep disorder group, with statistical significance (*P* < 0.01). Furthermore, 21 individuals (10.3%) in the sleep disorder group had psychological health issues, which was higher than the 11 individuals (3.2%) in the non-sleep disorder group, with statistical significance (*P* < 0.01). Refer to Table 3 for details.

**TABLE 3:** Comparison of participants’ sleep quality and sleep-related psychological scale data.

Item	PSQI ≤ 7 (*n* = 349)	PSQI>7 (*n* = 203)	χ^2^/Z	*P*-value
PSQI total score [points, (P25, P75)]	4.00 (3.00, 6.00)	10.00 (9.00, 12.00)	−19.677^△^	<0.001
Sleep onset score [points, (P25, P75)]	1.00 (1.00, 2.00)	3.00 (2.00, 3.00)	−13.050^△^	<0.001
Sleep duration score [(points, (P25, P75)]	0.00 (0.00, 1.00)	1.00 (1.00, 2.00)	−12.595^△^	<0.001
Sleep efficiency score [points, (P25, P75)]	0.00 (0.00, 1.00)	1.00 (0.00, 2.00)	−11.642^△^	<0.001
Sleep disturbances score [points, (P25, P75)]	1.00 (1.00, 1.00)	2.00 (1.00, 2.00)	−12.226^△^	<0.001
Sleep medication score [points, (P25, P75)]	0.00 (0.00, 0.00)	1.00 (0.00, 2.00)	−12.28^△^	<0.001
Daytime dysfunction score [points, (P25, P75)]	0.00(0.00,0.00)	1.00(0.00,2.00)	−9.206^△^	<0.001
Sleep quality score [points, (P25, P75)]	1.00 (1.00, 1.00)	2.00 (1.00, 2.00)	−15.338^△^	<0.001
ESS Score [points, (P25, P75)]	5.00 (2.00, 7.00)	6.00 (3.00, 9.00)	−4.825	<0.001
MEQ-5 Classification [*n* (%)]			18.159	0.001
Absolute evening type	3 (0.9)	4 (2.0)		
Moderate evening type	39 (11.2)	45 (22.2)		
Intermediate type	228 (65.2)	128 (63.0)		
Moderate morning type	77 (22.1)	25 (12.3)		
Absolute morning type	2 (0.6)	1 (0.5)		
GHQ-12 score [points, (P25, P75)]	0.00 (0.00, 1.00)	1.00 (0.00, 2.00)	−7.420^△^	<0.001
Daytime sleepiness (ESS ≥ 10 points) (%)	38 (10.9)	45 (22.2)	12.781	<0.001
Psychological health issues (GHQ-12 ≥ 4 points) (%)	11 (3.2)	21 (10.3)	12.160	<0.001

△ represents the Z value for the Mann-Whitney U test, all others represent χ^2^ values. PSQI, Pittsburgh Sleep Quality Index; ESS, Epworth Sleepiness Scale; MEQ-5, Morningness-Eveningness Questionnaire-5; GHQ-12, General Health Questionnaire-12.

### 3.4 *Logistic* regression analysis of risk factors for sleep disorders

Variables with statistical differences identified in the univariate analysis were included in the equation, and backward elimination was used for logistic regression analysis of sleep disorders. The results revealed that a history of previously diagnosed sleep disorders (OR 6.074, 95% *CI* 2.626–14.049, *P* < 0.001), rotating night shift work >3 times per week (OR 3.089,95%*CI* 1.650–5.781, *P* < 0.001), Using electronic devices > 1 h before sleep (OR 1.685,95%*CI* 1.131–2.511, *P* = 0.010), concern about contracting COVID-19 (OR 1.116, 95% *CI* 1.034–1.204, *P* = 0.005), ESS score (OR 1.068, 95% *CI* 1.018–1.121, *P* = 0.007), and GHQ-12 score (OR 1.511, 95% *CI* 1.281–1.782, *P* < 0.001) were identified as independent risk factors for healthcare workers experiencing sleep disorders. Conversely, a higher perception of societal support for supporting healthcare workers in Hubei (OR 0.861, 95% *CI* 0.744–0.998, *P* = 0.047) and a non-medical staff job role (OR 0.257, 95% *CI* 0.067–0.987, *P* = 0.048) were protective factors against sleep disorders. Refer to Table 4 for details.

**TABLE 4:** *Logistic* regression analysis of risk factors for sleep disorders.

Variable	Category	β Value	Wald	*P*-Value	OR (95% CI)
History of previously diagnosed sleep disorders	No	1.804	17.785	<0.001	6.074 (2.626∼14.049)
	Yes				
Weekly frequency of rotating night shift work	1 time				
	2 times	−0.179	0.386	0.534	0.836 (0.475∼1.472)
	3 times	0.083	0.076	0.782	1.087 (0.603∼1.959)
	> 3 times	1.128	12.434	<0.001	3.089 (1.650∼5.781)
Using electronic devices > 1 h before sleep	No				
	Yes	0.522	6.577	0.010	1.685 (1.131∼2.511)
Concern about contracting COVID-19		0.110	7.933	0.005	1.116 (1.034∼1.204)
Perception of societal support for supporting Hubei workers		−0.149	3.962	0.047	0.861 (0.744∼0.998)
Job role	Doctor				
	Nurse	0.308	1.308	0.253	1.361 (0.802∼2.310)
	Non-medical staff	−1.357	3.916	0.048	0.257 (0.067∼0.987)
ESS Score		0.066	7.292	0.007	1.068 (1.018∼1.121)
GHQ-12 Score		0.413	24.003	<0.001	1.511 (1.281∼1.782)

ESS, Epworth Sleepiness Scale; GHQ-12, General Health Questionnaire-12.

## 4 Discussion

During the COVID-19 pandemic, particularly in the early stages of the virus outbreak, healthcare workers often face numerous stressful events. These may include fear of an unknown virus, concerns about infection, shortages of protective equipment, long hours of intense and high-pressure work, rotating night shifts, and prolonged isolation, all of which can increase the psychological burden on healthcare workers (Ballesio et al., 2021). Unfortunately, during their support mission in Hubei, healthcare workers often experienced isolation in their daily lives, which could have adverse effects on their physical and mental health. Several reports have shown a significant increase in the incidence of sleep disorders, anxiety, and depression among frontline healthcare workers during the COVID-19 pandemic (Wang S. et al., 2020; Zhang et al., 2020). According to clinimetric criteria, when recent life events and/or chronic stressors are present, the body’s homeostatic maintenance system may be disrupted (Fava et al., 2019). Common stressors such as sleep disorders, daytime sleepiness, fatigue, anxiety, depression, and loss of will, continuously accumulate beyond individual coping levels, significantly affecting sleep and mental health (Fava et al., 2019). Particularly among individuals who have experienced isolation, emotional and sleep disturbances are more pronounced. Studies have shown that individuals working in a confined isolation environment are more likely to report fatigue, social withdrawal, anxiety when dealing with febrile patients, irritability, insomnia, lack of concentration, deterioration in work performance, reluctance to work, or considering resignation (Bai et al., 2004). Another study found that disease control measures such as quarantine may cause trauma to a significant proportion of children and parents. About 30% of isolated or quarantined children and 25% of isolated or quarantined parents met PTSD criteria, which is four times higher than the normal population (Sprang and Silman, 2013). Furthermore, the impact of the experience of confinement on the psychological health of the population may be prolonged. During the 2003 SARS epidemic, healthcare workers exposed to SARS showed increased levels of depression symptoms and sustained depression symptoms after the outbreak (Liu et al., 2012). Especially among those who have experienced isolation, it may lead to long-term poor mental health (Liu et al., 2012). Another study found that during isolation, the impact of economic income and the stigmatization of isolated patients by society could also exacerbate the psychological burden of isolation, leading to emotional and sleep problems (Brooks et al., 2020). Elderly individuals’ anxiety about contracting COVID-19 and potentially dying from it can also lead to adverse mental health outcomes (Hamidi et al., 2024; Namazi et al., 2024). Especially Oral and Maxillofacial Stress-Related Disorders will persist in the long term before and after COVID-19 infection (Ghasemzadeh-Hoseini et al., 2023). However, developing more reasonable and shorter isolation periods, providing as much information as possible, ensuring an adequate supply of basic supplies such as food, water, and medical supplies, avoiding loneliness, increasing communication (such as mobile phones, telephones, computers, psychological counseling team support, etc.), and widespread attention and support from society, as well as selfless dedication, can all improve the impact of isolation on the physical and mental health of healthcare workers (Brooks et al., 2020).

To the best of our knowledge, this is the first study on the sleep health of healthcare workers from Fujian Province supporting Hubei. Our study found that during the COVID-19 pandemic, the prevalence of sleep disorders among frontline healthcare workers was significantly higher than that of the general population (Sprang and Silman, 2013). The prevalence of sleep disorders reported in our study is similar to that reported by Bai et al. (2004). A study on the sleep status of Chinese nursing staff, which included 4951 clinical nursing staff, identified sleep disorders using a PSQI score > 5. The results showed that 63.9% of nursing staff had sleep disorders, and high night shift frequency and low social support were independently associated with sleep disorders (Liu et al., 2012). The results of these two studies may differ due to different scoring criteria. However, it is noteworthy that the prevalence of sleep disorders among frontline personnel is generally high, which will significantly affect their daily work and physical and mental health. Health authorities should focus on developing reasonable rest schedules and strengthening sleep health education to improve sleep quality, which will be beneficial for long-term epidemic prevention work. Our study also found a significant increase in daytime sleepiness among healthcare workers during the epidemic. However, interestingly, other surveys on daytime sleepiness among medical workers reported higher rates than our study (Brooks et al., 2020). We speculate that during the COVID-19 pandemic, frontline medical workers often experience fear of the virus due to lack of information about the source and treatment of the virus. When faced with major epidemics and emergencies, acute stress disorders are often widespread in the population (Huang and Zhao, 2020). Acute stress disorders often have symptoms such as excessive alertness, attention problems, excessive startle responses, and sleep disturbances (Dong et al., 2017). This can lead to excessive daytime awakening, reduced daytime sleepiness, but cannot maintain concentration, and may even increase the risk of nighttime sleep disturbances (Dong et al., 2017). Therefore, reasonable arrangements for daytime work, timely relaxation during daytime breaks, and strengthening understanding and education about the preventability and controllability of the virus among frontline medical staff can enhance their confidence. This will be beneficial for relieving their stress, daytime sleepiness, and nighttime sleep disturbances. A review of their sleep rhythms found that individuals with sleep disorders were more prominent in the evening. The study found that people with evening sleep rhythms were at increased risk of depression (Booker et al., 2018). Evening-type individuals are associated with later bedtimes and wake-up times, higher sleep requirements, more severe sleep deficits, morning sleepiness, and re-entry into sleep (Dutheil et al., 2021). Studies have also confirmed that eveningness is a strong predictor of poor sleep quality (Maercker et al., 2013), consistent with the results of this study. This suggests that adjusting previous sleep rhythms to avoid staying up late due to activities such as entertainment and chatting is beneficial for ensuring sleep quality. Particularly when facing major events, the habit of going to bed early and getting up early can directly help improve the sleep of frontline personnel.

*Logistic* regression analysis found that sleep disorders among healthcare workers were independently associated with a history of diagnosed sleep disorders, rotating night shifts more than 3 times per week, using electronic devices for more than 1 h before bedtime, concerns about COVID-19 infection, level of societal support for supporting Hubei medical workers, non-medical staff status, ESS score, and GHQ-12 score. Sleep disorders, especially insomnia, often have recurrent and chronic characteristics and significantly affect human physical and mental health. Therefore, the risk of developing sleep disorders is significantly increased in individuals with a history of diagnosed sleep disorders. When selecting frontline personnel for support, attention should also be paid to whether they have a history of sleep disorders, which is beneficial for the normal conduct of work. During the epidemic, due to heavy work tasks, healthcare workers often experience long-term sleep deprivation due to frequent rotating night shifts (Ballesio et al., 2021). Our study found a significant increase in the risk of sleep disorders when rotating night shifts more than 3 times per week, and other studies have also confirmed that frequent night shift rotations are a risk factor for sleep disorders (Liu et al., 2012). Using electronic devices for more than 1 h before bedtime is considered a risk factor for sleep disorders. The blue light from these devices may suppress melatonin secretion, causing neurophysiological arousal and affecting sleep (Romo-Nava et al., 2016). Prolonged use of mobile phones, especially when going to bed to sleep, can lead to prolonged sleep latency, low sleep efficiency, and daytime dysfunction (Taillard et al., 2004). Developing reasonable work schedules, reducing the frequency of night shifts, enriching activities outside of work, controlling the use of electronic devices, and sleep health education are helpful for sleep. The stress from the epidemic and the resulting anxiety can exacerbate concerns about COVID-19 infection, adversely affecting sleep (Megdal and Schernhammer, 2007). One of the observed relationships in our study is the impact of societal support on sleep disorders. Societal support plays a crucial role in mitigating stress and enhancing mental well-being. During the COVID-19 pandemic, various forms of societal support, such as community appreciation, financial incentives, and mental health resources, were provided to healthcare workers. These supports likely contributed to a reduction in sleep disorders by alleviating stress and providing emotional and psychological relief. Societal support for healthcare workers is a protective factor against sleep disorders, and the higher the level of support and understanding for healthcare workers, the lower the risk of sleep disorders (Megdal and Schernhammer, 2007; Liu et al., 2012), consistent with this study. Compared with frontline medical staff who need to face the tense treatment work and patients every day, non-medical staff have relatively fewer opportunities to contact patients, and the risk of sleep disorders is relatively lower (Sprang and Silman, 2013). Therefore, positive publicity at the national level, public opinion guidance, care and support from health authorities, and rational work scheduling will all help improve their sleep, giving them more confidence in overcoming the virus. Our study also found that ESS scores and GHQ-12 scores were independently associated with sleep disorders. The higher the score, the higher the likelihood of daytime sleepiness and psychological health problems, leading to an increased risk of sleep disorders, consistent with the results of the study by Brooks et al. (2020). All the results suggest that targeted interventions, such as providing robust mental health support and creating a supportive work environment, can significantly reduce the incidence of sleep disorders among healthcare workers. For instance, implementing regular mental health check-ins, offering counseling services, and ensuring adequate rest periods can help mitigate the impact of stress and improve sleep quality. Paying attention to the daily work status and mental health of frontline personnel, appropriate psychological interventions, and long-term psychological health follow-up interventions after the disaster should be actively carried out, both before and after the epidemic.

There are some limitations to this study. Firstly, due to the use of a cross-sectional study design and self-reporting methods, our study results cannot establish causal relationships. The study subjects were limited to healthcare workers from Fujian Province supporting Hubei, and due to different division of labor tasks in epidemic prevention and control work, they can only represent the sleep quality of regional healthcare workers. To draw conclusions about the sleep quality of all healthcare workers in China, further large-scale, multicenter studies are needed. Additionally, self-reported data can introduce response bias, where participants might over report or underreport their symptoms and behaviors. The use of the ESS scale, MEQ-5 scale, and GHQ-12 scale to detect sleepiness, sleep rhythms, and mental health status cannot be used to diagnose diseases such as narcolepsy, anxiety, and depression.

In summary, we observed that sleep disorders were very common among healthcare workers from Fujian Province supporting Hubei during the COVID-19 epidemic. A history of diagnosed sleep disorders, rotating night shifts more than 3 times per week, using electronic devices for more than 1 h before bedtime, excessive concern about COVID-19 infection, and poor psychological health were associated with an increased risk of sleep disorders, while a higher level of societal support and understanding for supporting Hubei medical workers was associated with a reduced risk of sleep disorders, and the risk of sleep disorders among non-medical staff was significantly reduced. Therefore, providing more sleep health education, psychological health services, and support for frontline healthcare workers during major events is necessary. Suggestions include: (1) Implementing a Psychological Support Program: Establishing a 24/7 psychological counseling hotline, organizing regular psychological health seminars, and providing online psychological support resources. (2) Developing More Reasonable Work Schedule Plans: Ensuring sufficient rest periods between shifts, limiting consecutive working hours, and offering flexible scheduling options. (3) Stress Management and Resilience Training: Conducting regular stress management workshops and offering online courses and tools to help healthcare workers cultivate skills to cope with high-pressure environments. (4) Sleep Health Education: Developing and implementing sleep health training programs to educate healthcare workers about early signs of sleep disorders and healthy sleep habits. Large-scale surveys of healthcare workers across the country are urgently needed.

## Data availability statement

The raw data supporting the conclusions of this article will be made available by the authors, without undue reservation.

## Ethics statement

The study was approved by the Ethics Committee of Fujian Provincial Hospital. All participants were informed and consented to participate in the survey.

## Author contributions

WY: Writing−review and editing, Writing−original draft, Visualization, Validation, Supervision, Software, Resources, Project administration, Methodology, Investigation, Funding acquisition, Formal analysis, Data curation, Conceptualization. LZ: Writing−original draft, Visualization, Supervision, Funding acquisition, Data curation, Validation, Software, Resources, Project administration, Methodology, Investigation, Formal analysis, Conceptualization. LY: Writing−review and editing, Writing−original draft, Supervision, Software, Methodology, Investigation, Data curation, Conceptualization, Visualization, Validation, Resources, Project administration, Funding acquisition, Formal analysis. LQ: Writing−review and editing, Writing−original draft, Visualization, Validation, Resources, Project administration, Funding acquisition, Formal analysis, Supervision, Software, Methodology, Investigation, Data curation, Conceptualization. XX: Writing−review and editing, Visualization, Validation, Software, Resources, Project administration, Investigation, Funding acquisition, Formal analysis, Conceptualization, Supervision, Methodology, Data curation. WS: Writing−original draft, Visualization, Validation, Supervision, Software, Resources, Project administration, Investigation, Funding acquisition, Formal analysis, Conceptualization, Methodology, Data curation.
